# Implementation of National Academies’ study recommendations: A fuel economy case study

**DOI:** 10.1073/pnas.2303242120

**Published:** 2023-03-28

**Authors:** Jennifer G. DiStefano, Elizabeth L. Zeitler

**Affiliations:** ^a^National Academies of Sciences, Engineering, and Medicine, Board on Energy and Environmental Systems, Washington, D.C. 20001

The National Academies of Sciences, Engineering, and Medicine (the Academies) serve the federal government, researchers, practitioners, and the public through thought leadership and authoritative, unbiased technical advice. Congress, federal agencies, and nonprofit or philanthropic organizations seek National Academies’ advice and expertise on energy and its environmental, economic, and social consequences, overseen by the Board on Energy and Environmental Systems (BEES). Recent BEES studies have addressed technology and policy for the electric grid (1), decarbonizing the energy system (2), evaluations of the Department of Energy’s research activities (3, 4), and assessment of the status of various energy technologies (5–8). To complete its studies, BEES assembles committees of experts with diverse backgrounds who meet, collect information, and come to consensus on a study report with findings and recommendations reflecting the committee’s conclusions. Committees operate independently of their sponsoring federal agency or other sponsor, with information gathering open to the public, and committee deliberations confidential.

Needs for advice change over time as the energy system and societal priorities evolve. Congress often mandates Academies’ studies when it authorizes new or modified action by a federal agency. For example, Congress has requested Academies’ advice on technologies for light-duty (passenger car and truck) vehicle fuel efficiency through mandates in 2001 (9) and 2007 (10). These mandates resulted in four consensus reports released in 2002, 2011, 2015, and 2021 (5, 11–13) sponsored by the Department of Transportation’s National Highway Traffic Safety Administration (NHTSA). These studies contained findings, recommendations, and detailed analyses that have helped to shape federal vehicle efficiency and greenhouse gas (GHG) emission regulations over the last two decades.

## Fuel Economy Standards Drive Energy Savings in the Transportation Sector

**Fig. 1.**
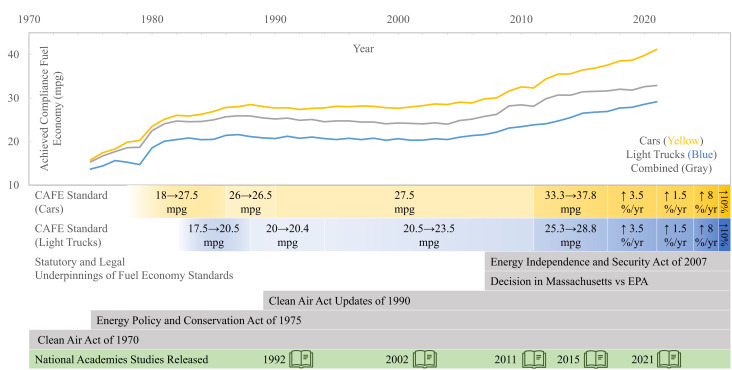
A history of the CAFE standards statutory and legal underpinnings (gray bars), operative standards for cars and light trucks (yellow and blue segments), National Academies’ studies (green bar), and fleet average regulatory compliance fuel economy for cars, light trucks, and all light-duty vehicles (yellow line, blue line, and gray line) in miles per gallon (mpg). Fuel economy data is from the EPA Automotive Trends report (17), and standards and legislative information is from the 2021 National Academies report (5).

Light-duty vehicle efficiency became particularly salient for policymakers and the public during the oil supply constraints and the resulting fuel price spikes in the mid-1970s. To reduce fuel consumption, Congress authorized NHTSA to regulate light-duty vehicle efficiency through Corporate Average Fuel Economy (CAFE) standards in the 1975 Energy Policy and Conservation Act (14) and subsequently updated including in the Energy Independence and Security Act of 2007 (10). As the urgency of addressing GHG emissions increased, the U.S. Environmental Protection Agency (EPA) also began to regulate vehicle GHG emissions under the Clean Air Act (15, 16).^*^ Fig. 1 presents the history of light-duty vehicle efficiency statutes, regulations, fleet-wide performance, and related Academies’ studies.

Vehicle fuel efficiency and GHG emission reduction have progressed dramatically since the first standards were established in 1978. CAFE/GHG standards both spurred technology development and were enabled by its progress. Technology advances included improved internal combustion powertrains, development of hybrid and alternative fuel vehicles (e.g., fuel cell and battery electric vehicles), and light-weighting and aerodynamic improvements. The technology, market, and regulatory environment has also been impacted by longer-lasting vehicles, a shift from passenger cars to crossovers and sport utility vehicles, regulations for criteria pollutants^†^ and safety, and international efficiency and GHG regulations. For example, emissions standards for health-harming criteria pollutants (e.g., NO_x_) motivated technology developments that have since been exploited for vehicle efficiency (17). From 1975 to 2020, the United States saw an approximately 110% improvement in fuel economy of new light trucks and approximately 150% improvement in fuel economy of new cars (Fig. 1) (18). It is estimated that only 15 to 20% of fuel economy savings^‡^ would have occurred without fuel economy regulations (19).

## Analysis of Academies’ Fuel Economy Recommendation Implementation

In this paper, we examine the record of Academies’ recommendations related to fuel economy for light-duty vehicles based on the Academies’ reports in 2002, 2011, and 2015, including topical areas covered and recommendation implementation status. An early report in 1992 (20) and a recently released 2021 report (5) are also referenced, though not evaluated for implementation, due to not having explicit recommendations (1992 report) or being too recent (2021 report).

To determine the implementation status of Academies’ recommendations, we primarily considered U.S. federal laws and regulations, focusing on NHTSA’s and EPA’s CAFE and GHG emission standards. Other publicly available information provided by the federal government (e.g., government program websites) was also referenced. Recommendations were categorized as implemented, partially implemented, uncertain, not implemented, or unevaluable. Recommendations classified as “uncertain” referred to information that would not necessarily reside in the public record (e.g., that the Agencies conduct research on topic or consult with certain stakeholders). Two recommendations were deemed “unevaluable” because they were not directed at a federal agency.

**Fig. 2.**
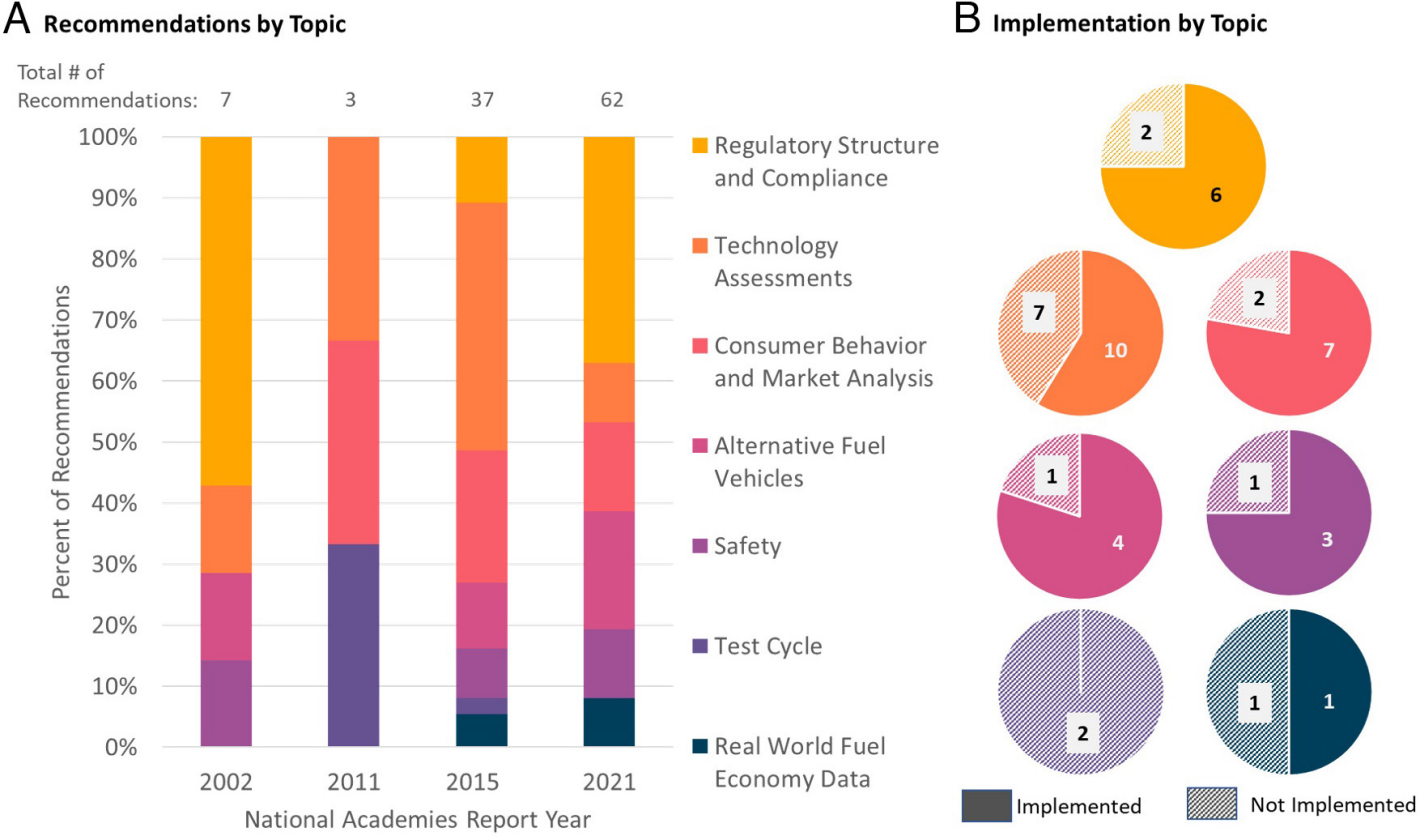
(*A*) Distribution of Academies’ recommendations by vehicle fuel economy topic for the 2002, 2011, and 2015 reports. The number of recommendations per report varies, so data are normalized for comparison. (*B*) Implementation record of Academies’ reports on fuel economy from 2002–2015 by topic. Recommendations that were fully or partially implemented are categorized as “Implemented,” and those that were not implemented or deemed uncertain or unevaluable are categorized as “Not Implemented.” Note that the implementation of the 2021 recommendations was not evaluated due to the recent report release.

Academies’ consensus study recommendations on vehicle fuel economy track the evolution of vehicle efficiency technology, markets, and policy (Table 1). Fig. 2*A* illustrates the evolving themes in the recommendations, shifting to address contemporary concerns around fuel economy technology and policy. Implementation assessment (Fig. 2*B*) documents the Academies’ impact on fuel economy regulations. The following discussion highlights a subset of these recommendations; a complete list of all recommendations evaluated and their implementation statuses can be found in *SI Appendix*.

**Table 1.** Summary of Academies’ recommendations from fuel economy technology assessment reports published in 2002, 2011, and 2015 in seven topic areas

Topic area	Summary of Academies’ recommendations from 2002 to 2015
Regulatory structure and compliance	• Fuel economy targets dependent on a vehicle attribute • Development of a single metric encompassing both GHG and petroleum reductions • Modifications to the test procedure • Understanding the role of automated vehicles on fleet fuel economy • Development and use of fuel economy credits, allowing trading of credits, and studying the effectiveness of CAFE and GHG credit markets
Technology assessment	• Identification of technologies in need of further or different analysis • Regulatory cost and effectiveness estimates including the specifics of the Agencies’ full system simulations and tear-down studies
Consumer behavior and market analysis	• Improved fuel economy labels for consumers • Analysis of consumer perceptions of fuel economy and resulting behavior • Examination of supply-side barriers and manufacturer actions in response to regulations • Changes to cost/benefit analyses
Alternative fuel vehicles	• Obtaining accurate cost and effectiveness estimates for AFVs, including recommending tear-down studies and investigations of battery life • Appropriate regulatory treatment of fuel consumption and emissions from AFVs
Safety	• Understanding the relationship between safety and fuel economy • Clarifying and mitigating safety risks in a fleet composed of traditional and light-weighted vehicles • Addressing concerns about trade-offs between tire safety and fuel economy
Test cycle	• Revising the test cycles to better reflect real-world operating conditions • Transitioning from the current two-cycle procedure to a five-cycle procedure
Real-world fuel economy data	• Data collection of on-road vehicle operation including fuel economy and other vehicle attributes to evaluate the effect of the CAFE and GHG standards and inform future test cycle requirements

## Analysis

### Technology Assessment.

Academies’ studies of light-duty vehicle fuel economy center on advanced vehicle technology advice to NHTSA. Requirements for agency technical analysis have increased over time due to government-wide obligations [i.e., Executive Order 12866 and the Regulatory Flexibility Act (21, 22)] and CAFE-specific requirements, including the mandate to establish the maximum feasible level of fuel economy (10). Academies’ studies provide extensive data and analysis of the cost and effectiveness of individual fuel economy technologies, many of which have been incorporated into NHTSA rulemakings. To augment these numeric estimates of cost and effectiveness, all Academies’ fuel economy studies have made recommendations on technology assessment. Recommendations on this topic have been implemented in 10 of 17 cases between 2002 and 2015, including adopting improved regulatory analysis methods (Fig. 2).

Between 2002 and 2021, Academies’ technology recommendations became more detailed and oriented toward regulatory analysis requirements, specifying particular technologies and methods. For example, the 2002 report offered no particular regulatory analysis suggestions and instead recommended funding precompetitive, breakthrough technology research to improve fuel economy, safety, and emissions. The 2011 report recommended vehicle-modeling techniques of full system simulation and vehicle system mapping for setting a regulatory baseline. In 2015 and 2021, extensive recommendations called for updated effectiveness and cost estimates through full system simulation and cost tear-down studies. Recommendations also suggested evaluation of gasoline octane level; battery size; and various aspects of technologies for spark ignition, electric, and diesel propulsion systems, vehicle design, and transmissions.

The use of vehicle full-system simulation, as recommended in the 2011 and 2015 reports, is one example of NHTSA implementing Academies’ advice and adopting improved analysis methods. These recommendations were implemented in the 2020 Safer Affordable Fuel-Efficient Vehicles Rule, via application of the Autonomie model developed by Argonne National Laboratory. The Agencies note that in order to implement this recommendation, they had to develop a large-scale simulation methodology to overcome the challenges of implementing full-system simulation on thousands of vehicle configurations (23).

### Treatment of Alternative Fueled Vehicles (AFVs).

AFVs have often been the subject of Academies’ recommendations, evolving with developments in technology, regulations, and markets. Over time, AFVs have progressed and proliferated from propane, methanol, and natural gas vehicles in the 1990s, to biofuel blend vehicles in the early 2000s, to the introduction of modern electric and hydrogen fuel cell vehicles in the 2010s. The 2002 report’s recommendations addressed biofuel AFVs and promotion of breakthrough AFV technologies. In the 2011 report, no recommendations on AFVs were made. By 2015, the report focused on hybrid, battery electric, and hydrogen fuel cell vehicles. The 2015 report included a review of specific electric powertrain technologies’ costs, capabilities, and safety aspects, and advice on the structure of the fuel economy program to cover alternative technology vehicles.

In the last few years, electric vehicles’ sales have begun to increase dramatically—and along with them—Academies’ recommendations on AFVs have grown and shifted to focus on electric and fuel cell vehicles. Plug-in electric vehicles’ sales’ rates rose from 0 to 1.5% of new vehicle sales between 2011 and 2017, to approximately 2% from 2018 to 2020, to over 4% in 2021, and over 6% in 2022 (through September) (24). As sales accelerated, AFV recommendations spiked in the 2021 report, with 27 recommendations addressing AFVs—more than the three other studies combined.^§^ The 2021 report’s first three summary recommendations highlighted AFVs’ importance, covering the growing role of zero-emission vehicles, the need for purchase subsidies for electric and fuel cell vehicles, and the need for vehicle charging infrastructure. Early signs indicate that some of the 2021 recommendations on electric vehicles are being implemented, such as extension and modification of federal purchase incentives for plug-in vehicles (25). Beyond electric vehicles, the repeatedly made Academies’ recommendation on accounting for actual use of biofuel blends in CAFE incentives was eventually implemented by the EPA and NHTSA (26).

### Real-World Fuel Economy Data.

Understanding real-world fuel economy is necessary to assess the impacts of the CAFE program and inform accurate testing procedures for regulatory compliance. Fuel economy test cycles are laboratory procedures used to measure vehicle compliance with fuel economy and emissions regulations, as proscribed in the Energy Policy and Conservation Act (14). The two test cycles, simulating highway and city driving, are known to overestimate fuel economy and exclude important factors relevant to real-world fuel economy, such as efficient air conditioning systems (19). This leaves little incentive for manufacturers to pursue the full breadth of fuel economy reduction routes (12, 13).^¶^ Further, the discrepancy between test cycle and real-world fuel economy has increased in recent years, which could degrade the impact of future fuel economy regulations (27).

The last comprehensive survey of vehicle fuel economy in the United States was in 1984. Since that time, new technologies and practices have become available to collect on-road fuel economy data. The 2015 Academies’ committee made two recommendations to the Agencies on real-world data collection: 1) to determine the adequacy of the current test cycle procedure and improve future test cycles, and 2) collect real-world data on fuel efficiency, vehicle footprint, fleet size mix, and price of new vehicles to better understand the effect of the CAFE/GHG standards and their impact on consumer and manufacturer choices. The recommendations were crafted acknowledging constraints of the EPCA test cycle requirements.

In the years after the 2015 recommendations, still no comprehensive data collection program exists for real-world fuel economy. Further, neither Congress nor the Agencies provided any updates on how test cycles are measured and used in the CAFE program. Therefore, the Academies were prompted to again publish multiple recommendations related to real-world fuel economy and test cycle effectiveness in their 2021 report. That committee recommended that the Agencies undertake a large-scale data collection effort on real-world fuel economy to improve the effectiveness of the CAFE program and research how well the test cycles reflect real-world driving. Based on the findings, the Academies recommend that the Agencies propose new vehicle test cycles.

### Safety.

All Academies’ reports on vehicle fuel economy have recognized the importance of understanding the relationship between fuel economy and safety. Vehicle light weighting is a key strategy to improve fuel economy but also raises concerns about safety in crashes. The impact of fuel economy technology on real-world safety is not simple to measure, however, due to the diverse circumstances that lead to, happen during, and result from crashes between different vehicles and their operators, pedestrians, and objects. Trends over time in vehicle masses, sizes, and technology adoption must be considered as new and legacy vehicles coexist on the road (13). Statistical analyses have shown that increasing the mass disparity of the fleet increases societal fatality risks; however, past studies may not describe the future fleet that is subject to new vehicle designs, new powertrain and safety technologies, and the adoption of CAFE standards that take into account vehicle footprint (5). The safety–fuel economy relationship is so important that it has even prompted a rare formal dissent in the 2002 report, about the uncertainty of the relationship, if any, between light-weighting and downsizing vehicles for fuel economy, and deaths in crashes (11).

In 2002, the Academies recommended that NHTSA further study the relationship between fuel economy and vehicle safety. In 2015, the Academies raised concerns about safety specific to a mixed fleet of old and new technology vehicles, recommending that NHTSA consider and mitigate negative impacts on safety arising during the transition period from the current vehicles to a more light-weighted fleet. Both recommendations were implemented by NHTSA (28). The National Academies, through the 2015 report, have also considered the relationship between tire safety and fuel economy. As recommended by the Academies, NHTSA has maintained the same tire safety regulatory system, which requires safety performance be maintained with fuel economy improvements.

The Academies continued to emphasize the importance of understanding and addressing safety in their 2021 report through several recommendations. Concerns about a mixed fleet were again raised; recommendations included that NHTSA should study changes in mass disparity from 2025 to 2035 and how these changes affect safety and that crash requirements address differences in vehicle size and mass. In total, seven recommendations in the 2021 report addressed safety, signifying the need for continued attention on this topic as individual vehicle technologies evolve and the fleet remains inhomogeneous.

## Conclusion

Fuel economy standards have been a centerpiece of U.S. energy policy since their introduction in 1978, contributing notably to energy security and sustainable transportation in the decades since. As Congress has increased stringency of fuel economy regulations and the complexity of analysis required, it has also increasingly asked the Academies to advise the federal government in this work. The analysis in this paper shows that the Agencies often, but not always, implement the recommendations of the Academies. In some cases, the Agencies cite Academies’ recommendations when updating their practices, indicating the influence of the recommendations on the regulatory process. Across the seven recommendation themes identified in this summary, suggested changes to regulatory test cycle was the only one where no recommendations have yet been implemented. Recommendations that are not implemented are often reiterated in a later report more specifically and directly. Across the 2002 to 2021 Academies’ reports, the 109 specific recommendations, 47 implementation statuses, and seven topical themes of regulatory structure and compliance, technology assessment, consumer behavior and market analysis, alternative fuel vehicles, safety, test cycle, and real-world fuel economy data provide a record of important fuel economy issues over time. This record of implementation can inform future work by the Academies, the Agencies, Congress, industry, and researchers as they examine, evaluate, and develop new fuel economy technologies, legislation, and policies.

## Footnotes

^*^Because they both have significant vehicle efficiency and emission regulations, NHTSA and EPA are collectively referred to as “the Agencies” in Academies fuel economy reports, in cases where both are relevant.

^†^Criteria pollutants are carbon monoxide, ground-level ozone, lead, nitrogen dioxide, particulate matter, and sulfur dioxide. They are harmful to human health and the environment and are regulated by the EPA under the Clean Air Act.

^‡^Analysis applies to fuel economy savings from 1975 through 2018.

^§^Note that this value includes both primary and secondary topical classification of recommendations. Only primary topic classification is considered in Fig. 2.

^¶^In 2008, the EPA adopted three additional test cycles to provide consumers with more accurate fuel economy information, as measured by CO_2_ emissions, via fuel economy labels, but these were not directly used for fuel economy compliance (27).
